# Copper Catalysis with Arynes: Unlocking Site-Selective Arylation of Pyrazoles

**DOI:** 10.21203/rs.3.rs-6323411/v1

**Published:** 2025-04-01

**Authors:** Vy Dong, Minghao Wang, Xintong Hou, Stephanie Corio, Lori Digal, Jennifer Hirschi

## Abstract

Arynes are among the most reactive species in organic chemistry—six-membered rings so strained that their energy rivals that of a hand grenade. ^1^ Since their discovery in 1902, chemists have used arynes to achieve innovative transformations and access diverse natural products, however, their application for catalytic cross-coupling remains unrealized. ^2^ A major challenge in late-stage functionalization is the selective *N* -arylation of unsymmetric pyrazoles to create a core found in blockbuster medicines worth over nineteen billion dollars annually. ^3^ Traditional cross-coupling methods usually favor one type of regioisomer and thus, limit late-stage access to alternatives that could speed up drug discovery. ^4,5^ Here, we show that copper catalysis harnesses arynes to achieve switchable arylation of pyrazoles. By tuning metallotautomers via ligand choice, we direct *N* -arylation to either nitrogen site in a pyrazole, unlocking site-selective control. ^6,7^ Mechanistic studies reveal how steric and electronic forces guide regioselectivity and turn an unpredictable process into a precise synthetic tool.

## Main text

For over a century, arynes have captivated chemists with their strained structure and unorthodox reactivity[1,8–10]—yet, the potential of arynes remains untapped in catalytic arylation of nitrogen—a cornerstone transformation in drug discovery.[5] In the meantime, the *N*-aryl pyrazole emerged as a pivotal pharmacore across blockbuster drugs for cancer, cardiovascular, and anti-inflammatory diseases–fueling the need for innovation in its construction.[3] De novo routes, such as the Knorr condensation with arylhydrazines, provide easy access to a wide-range of pyrazoles. However, regioselective control remains a drawback of this classic approach because a mixture of isomers is often obtained or access to only one isomer is possible depending on the substrate structure.[11] Preparation of parent pyrazoles followed by catalytic arylation would provide a powerful way to modify pyrazoles late-stage.[12] Well-established cross-couplings (Buchwald-Hartwig, Ullmann-Goldberg, Chan-Lam) typically favor the less hindered $N^{\beta}$, with isolated cases giving the opposite selectivity.[13] This general preference mirrors the innate selectivity of nucleophilic aromatic substitution ($S_NAr$), leaving the more crowded $N^{\alpha}$ site less accessible.[14–17] To rewrite this script, we propose harnessing arynes to arylate pyrazoles at the more elusive $N^{\alpha}$ site via Cu catalysis.[18,19]

Copper stands out as an attractive catalyst for development because of its low toxicity and high earth abundance.[20,21] While never isolated, Cu-aryne complexes have been detected in the gas-phase and implicated in aryne polymerizations, thus suggesting their viability as catalytic intermediates.[22,23] With unsymmetric pyrazole **1** and a Cu catalyst, we envisioned an equilibrium between metallotautomers **Cu-$N^{\beta}$** and **Cu-$N^{\alpha}$**, where either $N^{\beta}$ or $N^{\alpha}$ from the pyrazole coordinates to Cu, respectively (**Fig. 1**).[14] These Cu-pyrazolates intercept a transient aryne (**2**) formed in-situ and channel *N*-arylation through a five-centered transition-state. The design of this aminocupration draws insights from our recent discovery of a Cu-catalyzed enantioselective addition of pyrazoles to cyclopropenes.[18] Unlike arynes, cyclopropenes are stable and isolable, yet their high strain energy (54 kcal/mol) resembles that of fleeting aryne (50 kcal/mol).[24,25] This tenuous yet striking parallel led us to propose that Cu would not only orchestrate aryne activation with pyrazoles but exert control over regioselectivity. In general, orthogonal functionalization of heteroatoms with similar reactivity remains an important challenge,[4,5] however, examples of pyrazole alkylation has been reported.[26–29] We set out to identify Cu-catalysts that would ultimately allow unprecedented and switchable access to either $N^{\alpha}$-aryl pyrazole (**3**) or $N^{\beta}$-aryl pyrazole (**4**).

#### Reaction development and scope of $N^{\alpha}$-arylation

To begin our study, we chose 3-phenylpyrazole (**1a**) and commercial Kobayashi benzyne precursor (**2a,** *o*-(trimethylsilyl)phenyl triflate) to target regioselective formation of *N*-phenyl pyrazole **3a** (**Fig. 2**). Among the various aryne activation strategies, Kobayashi's mild protocol generates aryne using fluorides.[30] The control experiment in the absence of catalyst (entry 1) affords *N*-aryl pyrazoles as a 1:1 mixture of isomers in 75% yield, a facile and non-selective background reaction. We reasoned that it would be possible to outcompete this background transformation by using the appropriate Cu and ligand combination. From surveying ligands, we identified the Xantphos ligands as most promising. In particular, CyXantphos (**L1**) gives both high regioselectivity (10:1 $N^{\alpha}$:$N^{\beta}$) and reactivity (89%, entry 2). To further promote Cu-pyrazolate formation, we used organic and inorganic bases and found both to benefit selectivity, with TMG (1,1,3,3-tetramethylguanidine) providing over 20:1 $N^{\alpha}$:$N^{\beta}$ in selectivity and 92% in yield (entry 3). Similarly, $NEt_2$Xantphos (**L2**) furnishes an excellent ratio of **3a** to **4a** (>20:1), although with lower yield (47%, entry 4). In addition, we identified Josiphos (**L3-L4**) to be complementary ligands to Xantphos that also afford desirable reactivity and selectivity (entry 5-6). Increasing the amount of CsF results in lower selectivity presumably due to faster aryne formation which participates in the non-selective background transformation. Thus, matching the rate of aryne formation with Cu-aryne complexation results in a more selective arylation.

With the $N^{\alpha}$-conditions, we investigated the arylation of twenty-eight *N*-heterocycles (**1**) with benzyne precursor **2a**. **Figure 3A** highlights direct comparisons between literature reported examples and our arylation conditions. Through the use of Cu-diamine complexes, Buchwald provided seminal access to $N^{\beta}$-aryl pyrazoles under mild conditions.[14] Subsequent studies using both Cu and Pd also favor arylation of the less-hindered nitrogen.[31–33] In stark contrast, subjecting these exact pyrazoles to Cu-aryne catalysis generates the opposite constitutional isomers (**3o, 3r, 3s, 3t, 3w, 5d**). In addition to the reported substrates, we found excellent results with alkyl-substituted pyrazoles (**Fig. 3B, 3b-3e**). We observe an increase in regioselectivity as the alkyl substituent increases in size (4:1 to >20:1 $N^{\alpha}$:$N^{\beta}$). Halogen-containing pyrazoles (**3f, 3g**) exhibit modest yields (30-40%) and regioselectivities (5:1 to 10:1 $N^{\alpha}$:$N^{\beta}$). High yields (56-96%) and regioselectivities (13:1 to >20:1 $N^{\alpha}$:$N^{\beta}$) are obtained for 3-arylpyrazoles with substituents varying in electronics and sterics (**3h-3j, 3l-3p**), with the exception of 2-OMe (**3k**) and 3-OMe (**3p**). Heteroaryl substituents on pyrazoles, such as furyl (**3q**) and pyridyl (**3r**), also tolerate $N^{\alpha}$-arylation

(7:1 and >20:1 $N^{\alpha}$:$N^{\beta}$, respectively). To study more hindered substrates, we subjected various 3,5-disubstituted pyrazoles (**3s-3v**) to $N^{\alpha}$ conditions. Both regioselectivity and reactivity are maintained (67-90%, >20:1 $N^{\alpha}$:$N^{\beta}$). With an amino pyrazole substrate, hydroamination occurs at $N^{\alpha}$ rather than the amino group (**3w**, 58%, 7:1 $N^{\alpha}$:$N^{\beta}$). In the case of pyrazoles with 3-ester substituents, arylation at $N^{\beta}$ (positioned further away from the ester group) is favored (**3x**-**3y**, 1:9 to <1:20 $N^{\alpha}$:$N^{\beta}$). We postulate that chelation between the ester and $N^{\alpha}$ with Cu catalyst inverts selectivity to favor $N^{\beta}$-aryl pyrazoles. With an isomeric 4-ester pyrazole (where the ester group position prohibits chelation), we observe formation of the corresponding $N^{\alpha}$-aryl pyrazole **3z** (>20:1 $N^{\alpha}$:$N^{\beta}$). Extension to other important *N*-heterocycles provides both indazole (**3aa**) and triazoles (**3ab**, **3ac**). While arylation occurs favorably at the more-hindered $N^{\alpha}$ for indazole (when using dcypm ligand) and 1,2,4-triazole, the 1,2,3-triazole substrate tends to arylate at the middle nitrogen.[34]

Next, we apply the $N^{\alpha}$ protocol to ten substituted aryne precursors, including eight Kobayashi precursors (**Ary-I**) and two oxonium precursors (**Ary-II**) that can be activated by weak bases (**Fig. 3C**).[35] With Xantphos (**L1**-**L2**) and Josiphos ligands (**L3-L4**), we find that the $N^{\alpha}$-arylation of **1a** encompasses a variety of arynes (**2**) with different electronic and steric profiles. Symmetric benzynes and naphthalyne show high yields and regioselectivities to form C–N bonds with $N^{\alpha}$ (**5a-5c**, 50-76%, 11:1 to 16:1 $N^{\alpha}$:$N^{\beta}$). In the case of unsymmetric arynes, up to four possible constitutional isomers can be generated in one step because of both carbon and nitrogen site selectivity. For unsymmetric arynes, Garg and Houk established a distortion model to predict regioselective attack at the more distorted carbon sites.[36] But coordination of arynes with metals can alter this selectivity. Roberts demonstrated site-selective functionalization of arynes via the use of unsymmetrical ligands with Pd- or Ni-bound arynes to block one carbon site via sterics.[10,37] Here, we show in the absence of Cu-catalysts, pyrazole addition to an aryne bearing a methyl or trimethylsilyl group affords poor selectivity for both nitrogen and carbon sites (**5f and 5g**). In contrast, Cu catalyst (**L3**) promotes the addition with increased control for both the pyrazole and the aryne sites to generate **5f** (>20:1 $N^{\alpha}$:$N^{\beta}$, 1:5 $C^{1}$:$C^{2}$) and **5g** (>20:1 $N^{\alpha}$:$N^{\beta}$, <1:20 $C^{1}$:$C^{2}$). For an aryne bearing a phenyl group, we observe formation of **5h** (6:1 $N^{\alpha}$:$N^{\beta}$, <1:20 $C^{1}$:$C^{2}$), which favors the addition to the more hindered carbon site, in line with the catalyst-free conditions. For an electron deficient aryne (such as one bearing a nitro group, **5i**), we observe high selectivity for addition to the C1 position in line with that observed and predicted for substrate-control under the distortion model. While high nitrogen site selectivity is maintained with 4-substitued arynes (**5d, 5e**), the regiocontrol over carbon sites remains elusive due to the weak substituent effect.[30] Using free naphthalyne, we observe a mixture of isomers with a slight preference for C2 (1:2 $C^{1}$:$C^{2}$). But using Xantphos (**L1**) ligand, we were able to enhance the intrinsic carbon site selectivity (1:8 $C^{1}$:$C^{2}$) and provide improved $N^{\alpha}$-selectivity (**5j**). Surprisingly, by changing to Josiphos (**L4**) ligand, we observed a complete switch to favor the C1 site generating isomer **5j'** (6:1 $C^{1}$:$C^{2}$). In both cases, high nitrogen selectivity is observed. Together, these results highlight the promising ability of Cu-aryne catalysis to provide complementary control to Houk/Garg's use of free arynes and Robert's Pd/Ni-aryne complexes.

### Reaction development and scope of $N^{\beta}$-arylation

Compared to the control experiment (entry 1), addition of $Cu(MeCN)_4PF_6$ salt dramatically increases $N^{\beta}$-selectivity (entry 2). We sought to enhance this regio-control through ligands. Applying *rac*-BINAP increases the selectivity for $N^{\beta}$ (entry 3); the use of 1,10-phenanthroline (**L5**) affords excellent control (<1:20 $N^{\alpha}$:$N^{\beta}$) favoring **4a** (90%, entry 4).

Next, we illustrate arylation of $N^{\beta}$ by using complementary conditions featuring ligand **L5**. With alkyl substituted pyrazoles, we observe a similar trend in selectivity to $N^{\alpha}$ conditions. The regioselectivity increases in the order of 3-Me (**4b**, 1:9 $N^{\alpha}$:$N^{\beta}$), 3-cyclopropyl (**4c**, 1:12 $N^{\alpha}$:$N^{\beta}$), 3-*i*Pr (**4d**, <1:20 $N^{\alpha}$:$N^{\beta}$), and 3-*t*Bu (**4e**, <1:20 $N^{\alpha}$:$N^{\beta}$). Aryl- (**4f-4n**, 14-94%, 1:8 to <1:20 $N^{\alpha}$:$N^{\beta}$) and furyl- (**4o**, 81%, 1:13 $N^{\alpha}$:$N^{\beta}$) substituted pyrazoles afford the corresponding $N^{\beta}$-aryl product with moderate to excellent regioselectivities and yields. Compared to the $N^{\alpha}$ conditions, the regioselectivity of disubstituted pyrazoles shows a switch from the more hindered $N^{\alpha}$ to $N^{\beta}$ that has the smaller neighboring substituent (**4p-4s**, 55-98%, 1:8 to <1:20 $N^{\alpha}$:$N^{\beta}$). Indazole provides 40% yield and 1:10 regioselectivity towards the less-hindered $N^{\beta}$ (**4t**). We observe relatively poor compatibility with electron-deficient pyrazoles under $N^{\beta}$ conditions. In comparison, similar regioselectivity and higher yield are generally found under uncatalyzed reactions with these pyrazoles. Finally, to apply the orthogonal arylation conditions, we prepared a pyrazole-containing antipsychotic drug CDPPB (**7**) and its constitutional isomer CDPPB-*N*-isomer (**8**), containing either an $N^{\alpha}$- or $N^{\beta}$-phenyl motif.[38] While the known allosteric modulator **7** can be selectively constructed through the coupling between pyrazole **6** and benzyne precursor **2a** under $N^{\beta}$ conditions (56%, 1:6 $N^{\alpha}$:$N^{\beta}$), our $N^{\alpha}$ conditions switch the site selectivity and furnish arylation product **8** selectively (31%, 4:1 $N^{\alpha}$:$N^{\beta}$).

### Origin of regiodivergence: steric versus electronic ligand effects

To investigate the key regio-determining step, we prepared a Cu-pyrazolate (**CyXant-Cu-N$^{\alpha}$**) using mesitylcopper, **L1** and pyrazole **1a**.[21] This novel Cu-pyrazolate was isolated and characterized by X-ray crystallography and represents a rare mononuclear Cu(I) complex with non-bridging pyrazolate ligand.[39] Next, we subjected **CyXant-Cu-N$^{\alpha}$** to benzyne **2a** and identified formation of **3a**. In this experiment, we observed similar selectivity (14:1 $N^{\alpha}$:$N^{\beta}$ versus 20:1 $N^{\alpha}$:$N^{\beta}$) with slightly diminished efficiency (50% versus 92% yield) (**Fig. 5A**). This stoichiometric experiment supports the feasibility of Cu-pyrazolate as a viable intermediate in coupling with transient arynes.

To further probe the ligand effects on switchable site selectivity, we turned to density functional theory (DFT) calculations to model the reaction of unsymmetric 3-*t*-butyl-5-methyl pyrazole with benzyne precursor **2a** under $N^{\alpha}$- and $N^{\beta}$-conditions.[40] The lowest energy pathway for both ligand systems proceed through a 5-centered 1´,6´-migratory insertion aminocupration transition state.[18] There are two rapidly interconverting pyrazolate metallotautomers (Cu-$N^{\alpha}$ vs Cu-$N^{\beta}$). Consistent with the Curtin-Hammett principle, the product ratio is independent of the relative energies of the pyrazolate metallotautomers and is determined by the relative energies of the aminocupration transition states leading to each isomer (**Fig. 5B/5C**).[34] For the reaction of the unsymmetric pyrazole leading to **3t** ($N^{\alpha}$-aryl pyrazole) modeled with **L1** (CyXantphos), formation of the C–N bond (2.35 Å) is favored at the $N^{\alpha}$ position by 1.2 kcal mol (**TS-Nα-3t**, **Fig. 5B**), consistent with the experimentally observed regioselectivity (20:1 $N^{\alpha}$:$N^{\beta}$). Analysis of the key transition structures for the aminocupration steps indicates that the site selectivity is dictated by steric interactions between the *t*-butyl group and the bulky ligand, with the transition structure that avoids this steric clash (**TS-Nα-3t**) being lower in energy (see space filling models in **Fig. 5B**). In contrast, the less sterically demanding 1,10-phenanthroline ligand (**L5**) controls site selectivity via electronic factors, in particular by non-covalent dispersion interactions. The 1´,6´-migratory insertion transition structure that leads to the formation of the $N^{\beta}$ regioisomer of product **4q** displays net favorable dispersion interactions compared to **TS-Nα-4q** and is favored by 1.9 kcal/mol, consistent with the experimental regioselectivity (1:20 $N^{\alpha}$:$N^{\beta}$). Notable differences in these two transition structures are (1) the key C–N bond-forming distance (2.19 Å v/s 2.33 Å), and (2) the position of the *t*-butyl group relative to the phenanthroline p system; factors that dictate the extent of stabilization are derived from dispersive CH···N and CH–p interactions (see green areas in the dispersion maps in **Fig. 5C**). Therefore, regiodivergence is achieved by balancing the steric and electronic environment of the catalytic pocket — with bulkier ligands like **L1** directing $N^{\alpha}$-arylation, and less bulky ligands like **L5** permitting more favorable interactions within the pocket and thus favoring $N^{\beta}$-arylation.

#### Outlook

Over the past century, copper catalysis and aryne chemistry have advanced along separate tracks. Since Ullmann's stoichiometric C–N coupling in 1901 and the discovery of arynes in 1902, both fields have expanded the reach of synthetic methods.[21,41–44] Now, their merger opens a new path—copper-aryne catalysis uses ligands to control site-selective arylation. We focused on pyrazoles because of their vital role in medicine, and this study provides a switchable and site-selective strategy for C–N construction. However, the mechanistic insights from this study lay a foundation for future site- and stereoselective pyrazole transformations. As new precursors and conditions emerge, the chemistry of strained intermediates will only continue to advance. Given the vast toolbox of chiral ligands, this concept also sets the stage for enantioselective aryne chemistry, including cycloadditions, insertions, and beyond.

## Declarations

#### Acknowledgments

We are grateful to the NSF (CHE-1956457 for M.W., X.H., L.D., V.M.D.), the NIH (R35GM127071 for M.W., X.H., L.D., V.M.D. and R35GM147183 for S.A.C., J.S.H.). Computational support was provided through the ACCESS program, which is supported by National Science Foundation grants #2138259, #2138286, #2138307, #2137603, #2138296 utilizing allocations CHE180061 (J.S.H). We thank D. W. C. MacMillan

(Princeton) and R. G. Bergman (UC Berkley) for providing discussions in preparing the manuscript. We thank J. W. Ziller and L. M. Anderson-Sanchez of the UCI Crystallography Facility for X-ray crystallographic analysis. We thank UCI Mass Spectrometry Facility for assistance with HRMS. We also thank P. Wang, C. Becca and D. Zhou for performing experiments on the starting materials synthesis. M.W. thanks Y. Song for suggestions.

#### Author contributions

M.W., X.H., and L.D. designed, performed, and analyzed experimental studies. S.A.C. designed, performed, and analyzed computational studies. J.S.H. and V.M.D. supervised the project and manuscript preparation.

#### Competing interests

The authors declare that they have no competing interests.

#### Additional information

Crystallographic data for compound CyXant-Cu-$N^{\alpha}$ are available free of charge from the Cambridge Crystallographic Data Center under reference number CCDC-2384544. Supplementary Information is available for this paper.

Correspondence and requests for materials should be addressed to Vy M. Dong and Jennifer S. Hirschi.

## References

1. Wenk, H. H., Winkler, M. & Sander, W. One Century of Aryne Chemistry. *Angew. Chem. Int. Ed.* **42**, 502–528 (2003).
2. Tadross, P. M. & Stoltz, B. M. A Comprehensive History of Arynes in Natural Product Total Synthesis. *Chem. Rev.* **112**, 3550–3577 (2012).
3. Marshall, C. M., Federice, J. G., Bell, C. N., Cox, P. B. & Njardarson, J. T. An Update on the Nitrogen Heterocycle Compositions and Properties of U.S. FDA-Approved Pharmaceuticals (2013–2023). *J. Med. Chem.* acs.jmedchem.4c01122 (2024) doi:10.1021/acs.jmedchem.4c01122.
4. Blakemore, D. C. *et al.* Organic synthesis provides opportunities to transform drug discovery. *Nat. Chem.* **10**, 383–394 (2018).
5. Campos, K. R. *et al.* The importance of synthetic chemistry in the pharmaceutical industry. *Science* **363**, eaat0805 (2019).
6. Hartwig, J. F. Catalyst-Controlled Site-Selective Bond Activation. *Acc. Chem. Res.* **50**, 549–555 (2017).
7. Shugrue, C. R. & Miller, S. J. Applications of Nonenzymatic Catalysts to the Alteration of Natural Products. *Chem. Rev.* **117**, 11894–11951 (2017).

8. Hoye, T. R., Baire, B., Niu, D., Willoughby, P. H. & Woods, B. P. The hexadehydro-Diels–Alder reaction. *Nature* **490**, 208–212 (2012).
9. Niu, D., Willoughby, P. H., Woods, B. P., Baire, B. & Hoye, T. R. Alkane desaturation by concerted double hydrogen atom transfer to benzyne. *Nature* **501**, 531–534 (2013).
10. Humke, J. N. *et al.* Nickel binding enables isolation and reactivity of previously inaccessible 7-aza-2,3-indolynes. *Science* **384**, 408–414 (2024).
11. Ishihara, Y., Montero, A. & Baran, P. S. *The Portable Chemist's Consultant: A Survival Guide for Discovery, Process, and Radiolabeling.* (Apple Publishing Group, La Jolla, California, 2013).
12. Cernak, T., Dykstra, K. D., Tyagarajan, S., Vachal, P. & Krska, S. W. The medicinal chemist's toolbox for late stage functionalization of drug-like molecules. *Chem. Soc. Rev.* **45**, 546–576 (2016).
13. Li, F., Nie, J., Sun, L., Zheng, Y. & Ma, J. Silver-Mediated Cycloaddition of Alkynes with $CF_3CHN_2$: Highly Regioselective Synthesis of 3-Trifluoromethylpyrazoles. *Angew. Chem. Int. Ed.* **52**, 6255–6258 (2013).
14. Antilla, J. C., Baskin, J. M., Barder, T. E. & Buchwald, S. L. Copper–Diamine-Catalyzed *N*-Arylation of Pyrroles, Pyrazoles, Indazoles, Imidazoles, and Triazoles. *J. Org. Chem.* **69**, 5578–5587 (2004).
15. Ruiz-Castillo, P. & Buchwald, S. L. Applications of Palladium-Catalyzed C–N Cross-Coupling Reactions. *Chem. Rev.* **116**, 12564–12649 (2016).
16. West, M. J., Fyfe, J. W. B., Vantourout, J. C. & Watson, A. J. B. Mechanistic Development and Recent Applications of the Chan–Lam Amination. *Chem. Rev.* **119**, 12491–12523 (2019).
17. Ghosh, I. *et al.* General cross-coupling reactions with adaptive dynamic homogeneous catalysis. *Nature* (2023) doi:10.1038/s41586-023-06087-4.
18. Wang, M. *et al.* Copper-Catalyzed Hydroamination: Enantioselective Addition of Pyrazoles to Cyclopropenes. *J. Am. Chem. Soc.* **145**, 14573–14580 (2023).
19. Kim, K. & Lee, Y. Copper-Catalyzed Hydroamination of Oxa- and Azabenzonorbornadienes with Pyrazoles. *J. Org. Chem.* **87**, 569–578 (2022).
20. Bullock, R. M. *et al.* Using nature's blueprint to expand catalysis with Earth-abundant metals. *Science* **369**, eabc3183 (2020).
21. Kainz, Q. M. *et al.* Asymmetric copper-catalyzed C-N cross-couplings induced by visible light. *Science* **351**, 681–684 (2016).
22. Chai, Y., Shen, S., Weng, G. & Pan, Y. Gas-phase synthesis and reactivity of $Cu^+$–benzyne complexes. *Chem Commun* **50**, 11668–11671 (2014).
23. Mizukoshi, Y., Mikami, K. & Uchiyama, M. Aryne Polymerization Enabling Straightforward Synthesis of Elusive Poly(*ortho*-arylene)s. *J. Am. Chem. Soc.* **137**, 74–77 (2015).
24. Wiberg, K. B. The Concept of Strain in Organic Chemistry. *Angew. Chem. Int. Ed. Engl.* **25**, 312–322 (1986).
25. Kelleghan, A. V., Bulger, A. S., Witkowski, D. C. & Garg, N. K. Strain-promoted reactions of 1,2,3-cyclohexatriene and its derivatives. *Nature* **618**, 748–754 (2023).

26. Xu, D. *et al.* Magnesium-Catalyzed N2-Regioselective Alkylation of 3-Substituted Pyrazoles. *Synlett* **31**, 595–599 (2020).

27. Chen, S.-J., Golden, D. L., Krska, S. W. & Stahl, S. S. Copper-Catalyzed Cross-Coupling of Benzylic C–H Bonds and Azoles with Controlled *N*-Site Selectivity. *J. Am. Chem. Soc.* **143**, 14438–14444 (2021).

28. Desai, S. P., Yatzoglou, G., Turner, J. A. & Taylor, M. S. Boronic Acid-Catalyzed Regio- and Stereoselective *N*-Glycosylations of Purines and Other Azole Heterocycles: Access to Nucleoside Analogues. *J. Am. Chem. Soc.* **146**, 4973–4984 (2024).

29. Dalton, D. M., Walroth, R. C., Rouget-Virbel, C., Mack, K. A. & Toste, F. D. Utopia Point Bayesian Optimization Finds Condition-Dependent Selectivity for *N*-Methyl Pyrazole Condensation. *J. Am. Chem. Soc.* **146**, 15779–15786 (2024).

30. Shi, J., Li, L. & Li, Y. *o*-Silylaryl Triflates: A Journey of Kobayashi Aryne Precursors. *Chem. Rev.* **121**, 3892–4044 (2021).

31. Mukherjee, A., Subramanyam, U., Puranik, V. G., Mohandas, T. P. & Sarkar, A. Pyrazole-Tethered Heteroditopic Ligands and Their Transition Metal Complexes: Synthesis, Structure, and Reactivity. *Eur. J. Inorg. Chem.* **2005**, 1254–1263 (2005).

32. Xu, Z.-L. *et al.* Cu(OAc)2·H2O-catalyzed N-arylation of nitrogen-containing heterocycles. *Tetrahedron* **67**, 5282–5288 (2011).

33. Onodera, S., Kochi, T. & Kakiuchi, F. Synthesis of *N*-Arylpyrazoles by Palladium-Catalyzed Coupling of Aryl Triflates with Pyrazole Derivatives. *J. Org. Chem.* **84**, 6508–6515 (2019).

34. Ueda, S., Su, M. & Buchwald, S. L. Highly $N^2$-Selective Palladium-Catalyzed Arylation of 1,2,3-Triazoles. *Angew. Chem. Int. Ed.* **50**, 8944–8947 (2011).

35. Smith, O. *et al.* Harnessing triaryloxonium ions for aryne generation. *Nat. Synth.* (2023) doi:10.1038/s44160-023-00408-1.

36. Cheong, P. H.-Y. *et al.* Indolyne and Aryne Distortions and Nucleophilic Regioselectivites. *J. Am. Chem. Soc.* **132**, 1267–1269 (2010).

37. Denman, B. N., Plasek, E. E. & Roberts, C. C. Ligand-Induced Regioselectivity in Metal-Catalyzed Aryne Reactions Using Borylaryl Triflates as Aryne Precursors. *Organometallics* **42**, 859–864 (2023).

38. Lindsley, C. W. *et al.* Discovery of Positive Allosteric Modulators for the Metabotropic Glutamate Receptor Subtype 5 from a Series of *N*-(1,3-Diphenyl-1*H*- pyrazol-5-yl)benzamides That Potentiate Receptor Function in Vivo. *J. Med. Chem.* **47**, 5825–5828 (2004).

39. Watanabe, Y. *et al.* Copper(I)–Pyrazolate Complexes as Solid-State Phosphors: Deep-Blue Emission through a Remote Steric Effect. *J. Am. Chem. Soc.* **144**, 10186–10192 (2022).

40. M06-2X/6-311+G(d,p) PCM(acetonitrile)//M06-2X/6-31G(d) level of theory was employed for the shown models, as implemented in Gaussian 16 (52–56). See SI for comparison of key transition states at various levels of theory.

41. Gianatassio, R. *et al.* Strain-release amination. *Science* **351**, 241–246 (2016).

42. Wiesenfeldt, M. P. *et al.* General access to cubanes as benzene bioisosteres. *Nature* **618**, 513–518 (2023).
43. Kleinmans, R. *et al.* Intermolecular [2π+2σ]-photocycloaddition enabled by triplet energy transfer. *Nature* **605**, 477–482 (2022).
44. Tena Meza, A. *et al.* σ-Bond insertion reactions of two strained diradicaloids. *Nature* (2025) doi:10.1038/s41586-025-08745-1.
45. Hehre, W. J., Stewart, R. F. & Pople, J. A. Self-Consistent Molecular-Orbital Methods. I. Use of Gaussian Expansions of Slater-Type Atomic Orbitals. *J. Chem. Phys.* **51**, 2657–2664 (1969).
46. Miertuš, S., Scrocco, E. & Tomasi, J. Electrostatic interaction of a solute with a continuum. A direct utilizaion of AB initio molecular potentials for the prevision of solvent effects. *Chem. Phys.* **55**, 117–129 (1981).
47. Zhao, Y. & Truhlar, D. G. The M06 suite of density functionals for main group thermochemistry, thermochemical kinetics, noncovalent interactions, excited states, and transition elements: two new functionals and systematic testing of four M06-class functionals and 12 other functionals. *Theor. Chem. Acc.* **120**, 215–241 (2008).
48. Tomasi, J., Mennucci, B. & Cammi, R. Quantum Mechanical Continuum Solvation Models. *Chem. Rev.* **105**, 2999–3094 (2005).
49. Frisch, M. J.; Trucks, G. W.; Schlegel, H. B.; Scuseria, G. E.; Robb, M. A.; Cheeseman, J. R.; Scalmani, G.; Barone, V.; Petersson, G. A.; Nakatsuji, H.; Li, X.; Caricato, M.; Marenich, A. V.; Bloino, J.; Janesko, B. G.; Gomperts, R.; Mennucci, B.; Hratchian, H. P.; Ortiz, J. V.; Izmaylov, A. F.; Sonnenberg, J. L.; Williams; Ding, F.; Lipparini, F.; Egidi, F.; Goings, J.; Peng, B.; Petrone, A.; Henderson, T.; Ranasinghe, D.; Zakrzewski, V. G.; Gao, J.; Rega, N.; Zheng, G.; Liang, W.; Hada, M.; Ehara, M.; Toyota, K.; Fukuda, R.; Hasegawa, J.; Ishida, M.; Nakajima, T.; Honda, Y.; Kitao, O.; Nakai, H.; Vreven, T.; Throssell, K.; Montgomery, J. A., Jr.; Peralta, J. E.; Ogliaro, F.; Bearpark, M. J.; Heyd, J. J.; Brothers, E. N.; Kudin, K. N.; Staroverov, V. N.; Keith, T. A.; Kobayashi, R.; Normand, J.; Raghavachari, K.; Rendell, A. P.; Burant, J. C.; Iyengar, S. S.; Tomasi, J.; Cossi, M.; Millam, J. M.; Klene, M.; Adamo, C.; Cammi, R.; Ochterski, J. W.; Martin, R. L.; Morokuma, K.; Farkas, O.; Foresman, J. B.; Fox, D. J. Gaussian 16; Rev. C.01: Wallingford, CT, 2016.
50. Lu, T. & Chen, Q. Independent gradient model based on Hirshfeld partition: A new method for visual study of interactions in chemical systems. *J. Comput. Chem.* **43**, 539–555 (2022).

## Figures

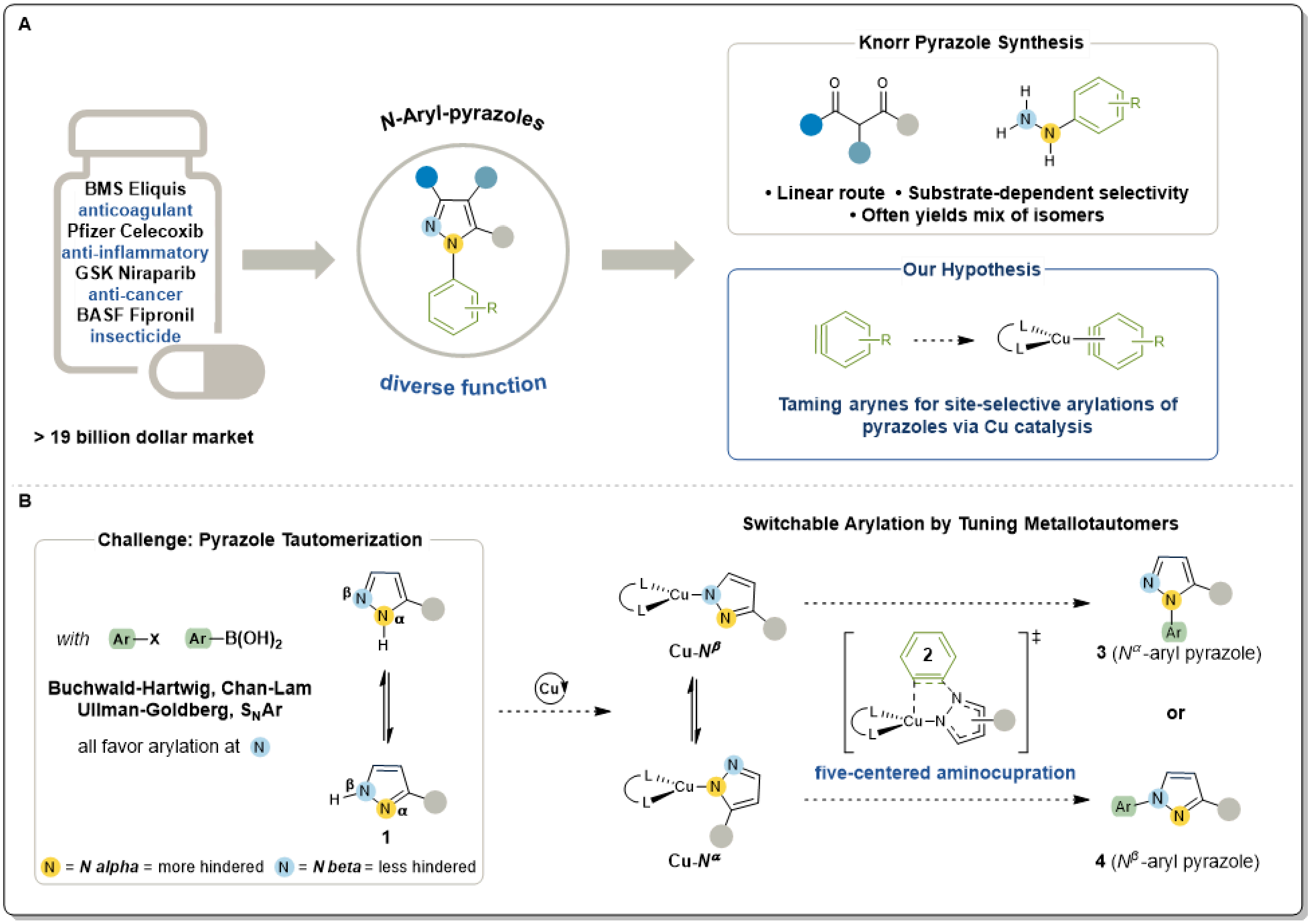

### Figure 1

**Background and our design. a**, *N*-aryl pyrazoles represent common motifs in medicines. Main drawback of the Knorr pyrazole synthesis is substrate-dependent or lack of regiocontrol. We propose Cu-aryne catalysis for achieving late-stage pyrazole arylations. **b**, Pyrazoles exist as tautomers where the less-hindered $N^{\beta}$ site undergoes arylation via traditional cross-coupling. We propose that tuning the ligands in metallotautomers will enable a new and switchable arylation for $N^{\alpha}$ or $N^{\beta}$ sites.

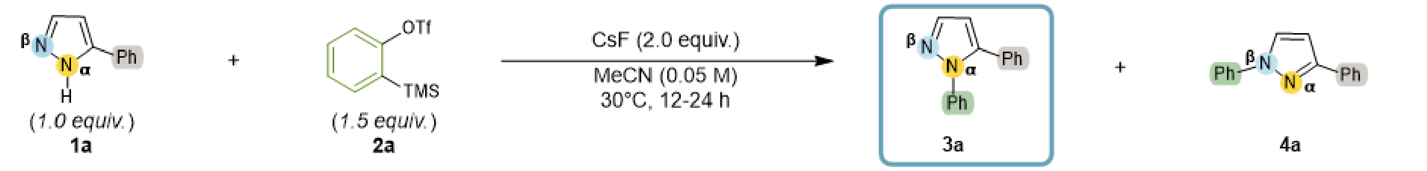

| entry | variation | $N^{\alpha}$:$N^{\beta}$ (**3a**:**4a**) | yield |
|---|---|---|---|
| 1 | none | 1:1 | 75% |
| 2 | with $Cu(CH_3CN)_4PF_6$ (10 mol%) + L1 (12 mol%) | 10:1 | 89% |
| **3** | **with $Cu(CH_3CN)_4PF_6$ (10 mol%) + L1 (12 mol%) + TMG (0.5 eq)** | **>20:1** | **92%** |
| **4** | with $Cu(CH_3CN)_4PF_6$ (10 mol%) + L2 (12 mol%) + TMG (0.5 eq) | >20:1 | 47% |
| 5 | with $Cu(CH_3CN)_4PF_6$ (10 mol%) + L3 (12 mol%) + TMG (0.5 eq) | >20:1 | 81% |
| 6 | with $Cu(CH_3CN)_4PF_6$ (10 mol%) + L4 (12 mol%) + TMG (0.5 eq) | 7:1 | 75% |

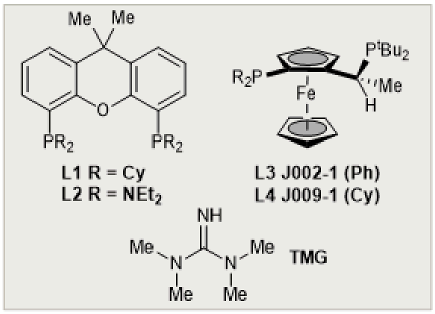

### Figure 2

**Reaction optimization of $N^{\alpha}$-selective arylation.** Reactions were performed on a 0.050 mmol scale. Yield and regioselectivity ($N^{\alpha}$:$N^{\beta}$) were determined by $^1$H NMR analysis of the reaction mixture.

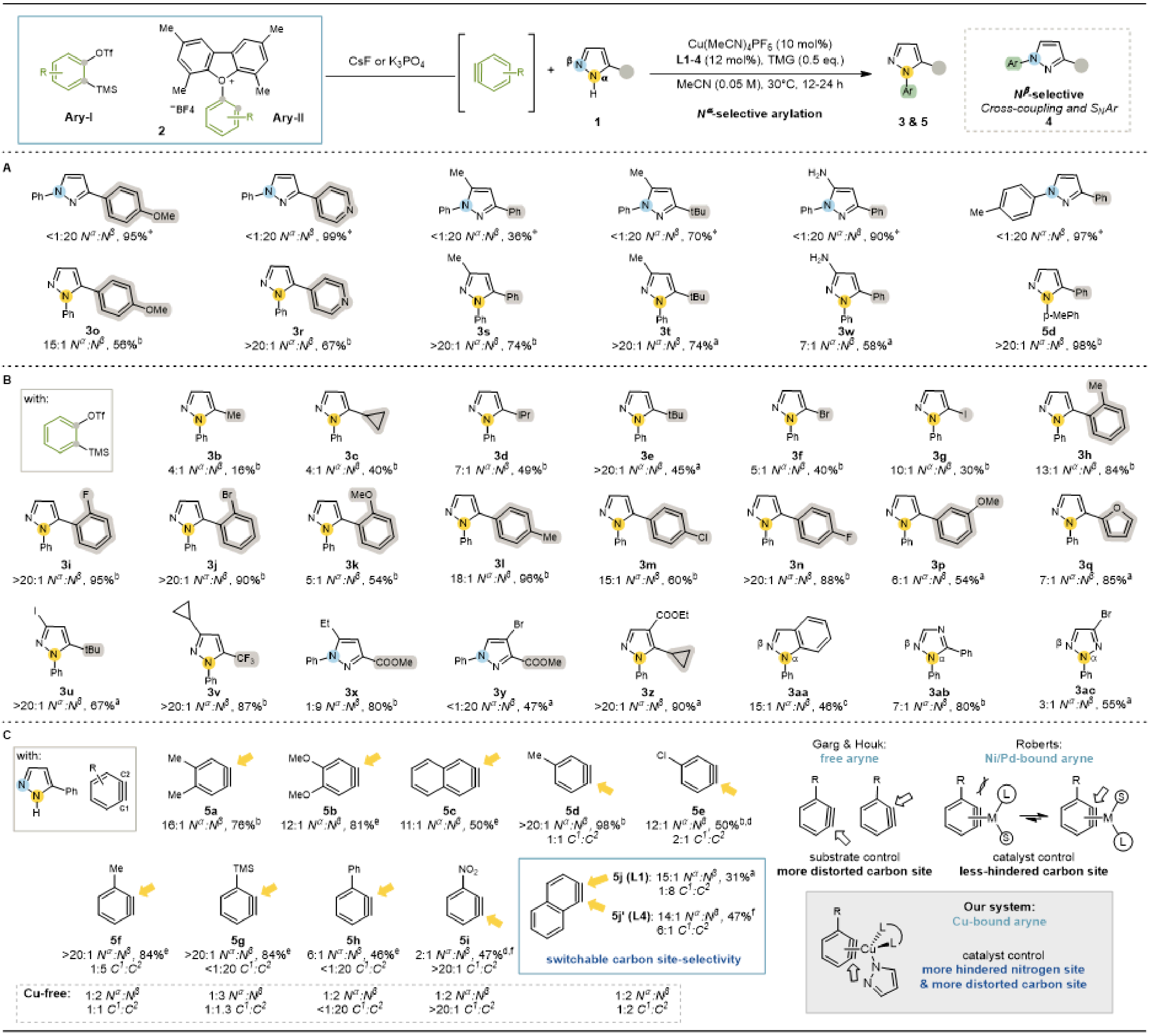

### Figure 3

**$N^{\alpha}$-arylation. a**, Comparing our chemistry with reported cross-couplings. **b**, Scope of pyrazoles. **c**, Scope of arynes and state-of-the-art models for predicting aryne regioselectivity. Standard reaction conditions: **1** (0.050 mmol), **Ary-I** (0.075-0.15 mmol), $Cu(CH_3CN)_4PF_6$ (10 mol%), **L** (12 mol%), TMG (50 mol%), CsF (2.0-4.0 equiv.), MeCN (1.0 mL), 12-24 h, 30 °C. [a]With **L1**. [b]With **L2**. [c]With bis(dicyclohexylphosphino)methane (dcypm) ligand. [d]With **Ary-II** (0.075mmol), $K_3PO_4$ (2.0 equiv.) instead. [e]With **L3**. [f]With **L4**. Isolated yield of **3** and **5**. Regioselectivty ($N^{\alpha}$:$N^{\beta}$) was determined from $^1$H NMR analysis of the reaction mixture. See Supplementary Information for full reaction conditions for each substrate.

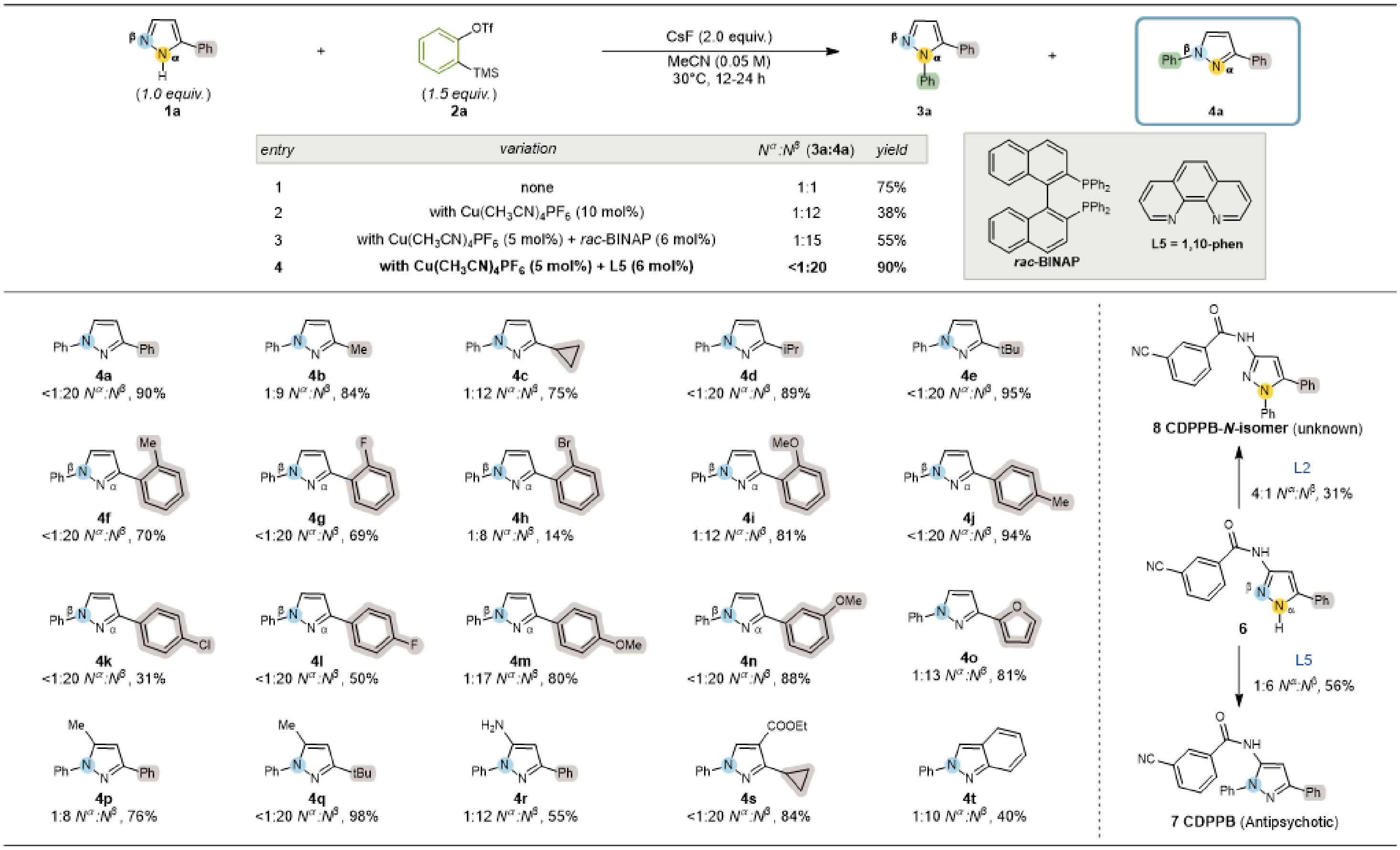

### Figure 4

**Reaction optimization of $N^{\beta}$-selective arylation and scope.** Standard reaction conditions: **1** (0.10 mmol), **2a** (0.15-0.30 mmol), $Cu(CH_3CN)_4PF_6$ (5 mol%), **L5** (6 mol%), CsF (2.0-4.0 equiv.), MeCN (0.4 mL), 12-24 h, 30 °C. Isolated yield of **4**. Regioselectivty ($N^{\alpha}$:$N^{\beta}$) was determined from $^1$H NMR analysis of the reaction mixture. See Supplementary Information for full reaction conditions for each substrate.

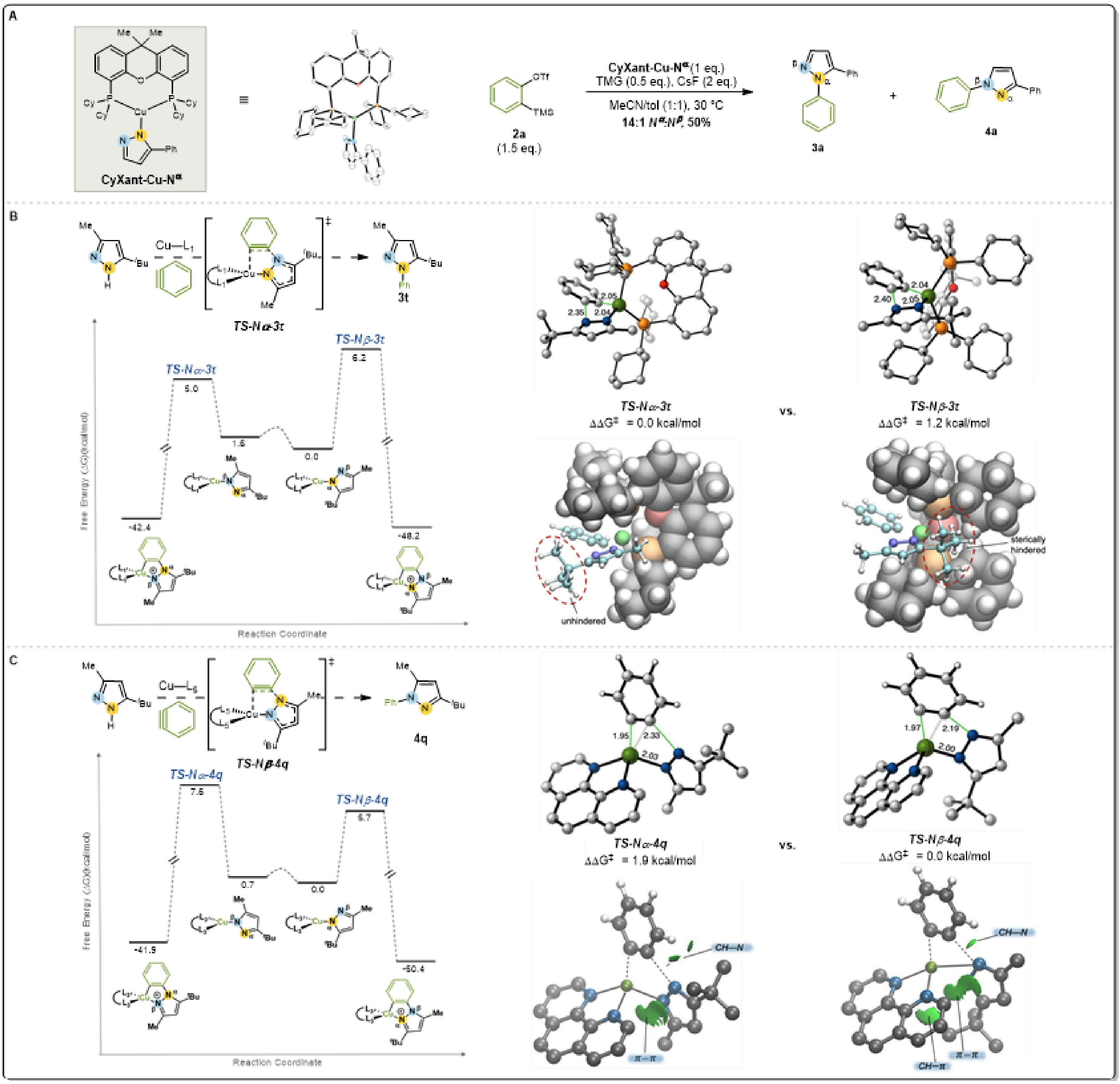

### Figure 5

**Origin of site-seletivity. a**, Synthesis of Cu-pyrazolate metallo-tautomer and stochiometric reactivity. **b**, Steric-controlled regioselectivity. For ligand **L1** (Cy-Xantphos), shown are: (*left*) energy diagram depicting the relative barriers of the regiodivergent aminocupration TSs relative to the pyrazolate tautomers; (*top right*) structure of the lowest energy TSs of the 5-centered aminocupration step leading to both $N^{\alpha}$- and $N^{\beta}$-arylation at M06-2X/6-311+G(d,p) PCM (acetonitrile)//M06-2X/6-31G(d) level of theory;[45–49] (*bottom right*) space filling models illustrating the steric constraints of each TS. **c**, Electronic-controlled regioselectivity. For ligand **L5** (1,10-Phenanthroline), shown are: (*left*) energy diagram depicting the relative barriers of the regiodivergent aminocupration TSs relative to the pyrazolate tautomers; (*top right)* lowest energy TSs of the 5-centered aminocupration step leading to both $N^{\alpha}$- and $N^{\beta}$-arylation; (*bottom right*) non-covalent interaction (NCI) plots depicting intermolecular dispersion interactions (shown in green) between pyrazole and Cu-**L5** and **2a** (isosurface 0.009).[50]

## Supplementary Files

This is a list of supplementary files associated with this preprint. Click to download.

- SupplementaryInformation.docx
